# The effect of Hp infection on dyslipidemia in Asia and out of Asia: a systematic review and meta-analysis

**DOI:** 10.3389/fmed.2025.1643218

**Published:** 2025-10-16

**Authors:** Kong-jin Quan, Zhen-peng Huang, Sha Nie, Xiao-xiao Li

**Affiliations:** Faculty of Nursing, Guangxi University of Chinese Medicine, Nanning, China

**Keywords:** *Helicobacter pylori*, infections, dyslipidemia, systematic review, meta-analysis

## Abstract

**Introduction:**

Helicobacter pylori (Hp) infection is a major public health problem worldwide. Similarly, millions suffer from dyslipidemia, which is a risk factor for cardiovascular and cerebrovascular diseases. This study aimed to explore whether Hp infection affects dyslipidemia.

**Methods:**

The search databases included PubMed, Web of Science, Cochrane Library, Embase, China National Knowledge Infrastructure, Wanfang Data, Chinese sci-tech periodicals, and Sino-Med, from database construction to September 2024. Casecontrol and cross-sectional studies on Hp infections associated with dyslipidemia were included.

**Results and Discussion:**

In total, 12 casecontrol and 28 cross-sectional studies were included. The total sample size included 274,414 cases, of which 130,529 were Hp-infected and 143,885 were uninfected. Compared to uninfected patients, Hp-infected patients abnormally elevated total cholesterol (TC) [Mean Difference (MD) = 0.15, 95% Confidence Interval (CI) 0.130.17, *p* < 0.05], low- density lipoprotein cholesterol (LDL-C) [MD = 0.18, (95%CI 0.140.22), *p* < 0.05]; TG [MD = 0.13, (95%CI 0.100.16), *p* < 0.05], and triglyceride (TG) levels and abnormally reduced high-density lipoprotein cholesterol (HDL-C) levels [MD = 0.02, (95%CI 0.03 to 0.01), *p* < 0.05]. Hp infection was correlated with dyslipidemia, and the effect of Hp infection on dyslipidemia varies in different regions.

**Systematic Review Registration:**

identifier CRD42024626356.

## Introduction

*Helicobacter pylori* (Hp) was first isolated in 1982 by Marshall and Warren from gastric biopsy specimens of patients with chronic gastritis (1). Many studies have shown that Hp infection can not only trigger gastrointestinal diseases such as chronic gastritis, gastric ulcer, duodenal ulcer, gastric cancer, and malignant lymphoma of the gastric mucosa but may also be associated with the pathogenesis of non-gastric diseases such as Alzheimer’s disease, Parkinson’s disease, and atherosclerosis (2–4). Hp has been identified as a class I carcinogen by the International Agency for Research on Cancer, and approximately 90% of distal gastric cancers are attributable to Hp infection (5). Hp infection remains a major public health problem worldwide, with the crude global prevalence of Hp in adults estimated to be 43.9% in the year 2022, and the prevalence of Hp infection in Africa, the Eastern Mediterranean region, and Southeast Asia is estimated to be 52.7, 52.6, and 46.7%, respectively (6). The prevalence of Hp infection varies across countries depending on age, ethnicity, geographic region, socioeconomic status, and hygiene conditions (7).

Dyslipidemia refers to abnormalities in lipoprotein metabolism, including elevated levels of total cholesterol (TC), low-density lipoprotein cholesterol (LDL-C), and triglycerides (TG), and/or reduced high-density lipoprotein cholesterol (HDL-C) (8). Approximately 220 million people worldwide suffer from dyslipidemia, which results in 4 million deaths annually (8). Dyslipidemia is one of the most important factors leading to atherosclerosis and is an independent risk factor for coronary heart disease and ischemic stroke (9, 10). The global prevalence of hypertriglyceridemia in adults aged 25 years and above is about 39%, and abnormally elevated plasma LDL-C levels have become the 8th leading risk factor for death (11, 12). The prevalence of dyslipidemia varies across countries and regions around the world. Plasma hypertriglyceridemia affects approximately 20% of Kazakhstan’s total population (13). The prevalence of dyslipidemia in China is 33.8% (14). Approximately 80% of the adults in Turkey have at least one lipid abnormality (15), as have 67% of adults in Romania. Meanwhile, approximately 77.2% of Polish adults suffer from dyslipidemia (16, 17).

Recent studies have debated whether Hp infection affects dyslipidemia. It has been suggested that Hp infection may trigger a chronic inflammatory response, which has been associated with dyslipidemia in some studies (18–21). In contrast, other studies have concluded no clear and stable correlation exists between Hp infection and dyslipidemia (22, 23). Findings regarding the relationship between Hp infection and dyslipidemia are conflicting, and an in-depth exploration of these controversial studies is of great significance in clinical practice. Clarifying whether Hp infection has an impact on dyslipidemia will not only help clinicians develop more targeted treatment strategies, but also guide early screening and intervention in high-risk groups, helping patients receive more personalized and effective treatment plans, and ultimately improving their lipid control and overall health prognosis. Besides, there are limited studies on the relationship of Hp infection on dyslipidemia in different regions. Therefore, more high-quality research to clarify the link between the two is crucial. This study collected relevant data on the relationship between Hp infection and dyslipidemia and analyzed whether Hp infection affects dyslipidemia.

## Materials and methods

This systematic review was conducted following the Preferred Reporting Items for Systematic Review and Meta-analysis (PRISMA) statement and was registered in the International prospective register of systematic reviews (CRD42024626356) (24).

### Databases and search strategy

The computerized search databases included PubMed, Web of Science, Cochrane Library, Embase, China National Knowledge Infrastructure, Wanfang Data, Database of Chinese sci-tech periodicals, and Sino-Med. The data collection period was from the construction of the database to September 2024. This study employed search terms including “*Helicobacter pylori*,” “Helicobacter Infections,” “Dyslipidemias,” and “Dyslipidemia.” And used a combination of subject lines and free words and Boolean logical operators such as “AND,” “OR,” and “NOT” to combine search terms to form a search formula. The search strategy was customized for each database. The PubMed search strategy was used as an example (Supplementary Table 1).

### Inclusion and exclusion criteria

Inclusion and exclusion criteria were determined according to the PECOS principles. The inclusion criteria were defined as follows: For the Population (P), the study included adults over 18 years of age diagnosed with Hp infection. No restrictions were applied regarding sex or ethnicity. For Exposure (E), Hp infection status served as the primary exposure factor. For the Comparator (C), healthy control groups comprised individuals without Hp infection, matched by age and sex. For the Outcome (O), indicators included plasma levels of TC, TG, LDL-C, and HDL-C, to compare these levels between Hp-infected participants and non-infected controls. Concerning the Study design (S), all types of primary research exploring the association between Hp infection and dyslipidemia were included, specifically cross-sectional studies, case–control studies, and cohort studies. No restrictions were placed on publication year, language, or Hp detection method criteria.

The exclusion criteria were as follows: For the Population (P), individuals receiving anti-Hp therapy, lipid-lowering therapy, or antibiotic therapy were excluded. For Exposure/Comparison (E/C), participants with comorbidities known to affect lipid metabolism-namely, coronary heart disease, diabetes mellitus, metabolic syndrome, severe liver or kidney disease, or malignant tumors-were excluded. Individuals under 18 years of age were also excluded based on the P criterion. For the Outcome (O), studies lacking essential data or for which such data were unavailable were excluded. Regarding the Study design (S), literature that could not be retrieved in full, along with secondary research types (e.g., reviews, meta-analyses), conference abstracts, academic reports, guidelines, protocols, animal studies, and cellular experiments, were excluded. The inclusion and exclusion criteria are shown in Supplementary Table 2.

### Literature screening and data extraction

After removing duplicates from all studies using EndNote X9 software, two evaluators (Qkj and Lxx) independently screened study titles and abstracts related to the relationship between Hp infection and dyslipidemia and screened the entire study based on the inclusion and exclusion criteria. Both screening rounds were conducted under the guidance of a third reviewer (Hzp).

Two reviewers (Qkj and Lxx) independently extracted the relevant data from the included studies. Extracted information from literature was as follows: First author, Year of publication, Country, Research type, Sample size, Hp detection methods, Lipid levels in Hp-positive (mmol/L, $\bar{x}\pm s$), Lipid levels in Hp-negative (mmol/L, $\bar{x}\pm s$), findings.

### Literature quality evaluation

Two reviewers independently assessed the risk of bias in the included studies (Qkj and Lxx). Evaluation results were compared, and if consensus could not be reached, the decision was made by the third reviewer (Hzp) or through group discussion. The Australian Joanna Briggs Institute Centre for Evidence-Based Health Care’s Realistic Evaluation Tool for Case–Control Studies consists of 10 evaluation items: (1) comparability of cases and controls apart from exposure; (2) appropriate matching between cases and controls; (3) use of the same criteria for the recruitment of cases and controls; (4) use of standard, valid, and reliable methods to measure exposure; (5) use of the same methods to measure exposure in both cases and controls; (6) consideration of confounding factors; (7) control of confounding factors; (8) use of standard, valid, and reliable methods to measure outcomes; (9) adequacy of the exposure duration; and (10) appropriate statistical methods for data analysis? Each item was evaluated by answering “Yes,” “No,” “Unclear,” or “Not applicable” (25).

### Statistical methods

Meta-analysis was performed using Review Manager 5.4 and Stata 15.1. According to the Cochrane Handbook for Systematic Reviews of Interventions (version 6.5), model selection is no longer determined by the magnitude of heterogeneity (26). Given the multiple sources of clinical heterogeneity in the literature included in this study, such as age distribution, sex ratio, body mass index, geographic characteristics, and research type, a random-effects model was used for meta-analysis, with effect sizes expressed as the MD and its 95%CI. Statistical significance was set at *p* < 0.05.

This study assessed the impact on the total effect size using the leave-one-out method from sensitivity analysis. Sources of heterogeneity were explored using predetermined factors, such as region, country, research type, and our interpretation was based on a *p*-value threshold of 0.05. When subgroup analyses suggested that heterogeneity might be influenced by covariates, meta-regression was used to analyze the ratios of sex, average age, and BMI to quantify the association strength. Funnel plots supplemented with Egger’s and Begg’s tests were used to detect publication bias and to explore the stability of the findings. Egger’s and Begg’s tests with *p*-values greater than 0.05 suggested significant publication bias did not exist. If asymmetry existed, effect sizes were corrected using the trim and fill method.

## Results

### Results of study screening

**Figure 1:**
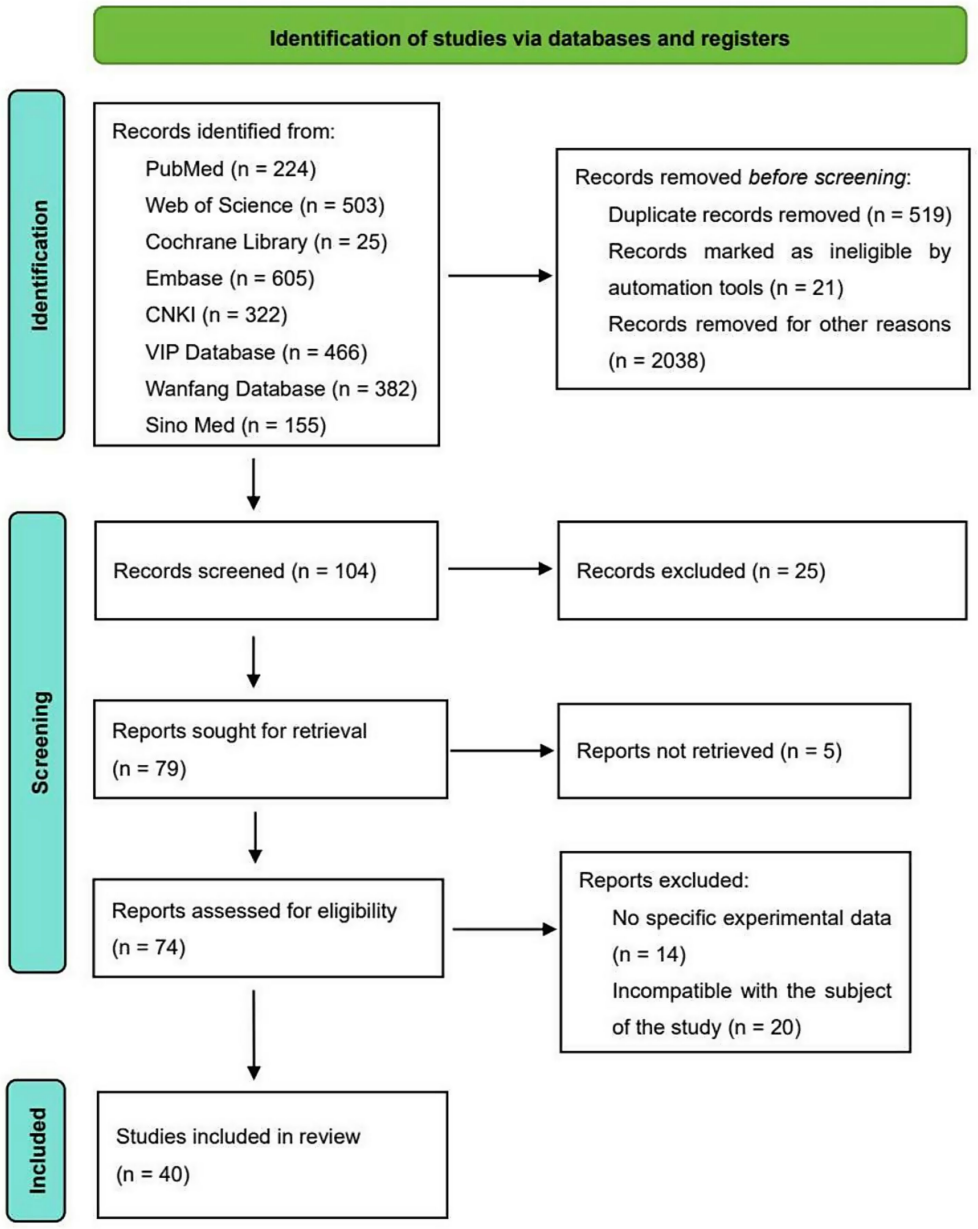
Flow diagram of literature screening.

The initial search yielded 2,682 studies, of which 40 were included. The screening process and results are shown in Figure 1.

### Characteristics of included studies

The included studies were published between 2003 and 2024 with a total sample size of 274,414 cases, including 130,529 Hp-infected and 143,885 Hp-uninfected cases (18–21, 27–62). In total, 12 case–control and 28 cross-sectional studies were included. The characteristics of the included studies are shown in Supplementary Table 3.

### Methodological quality assessment of the included studies

The quality of the studies was assessed using the Australian Joanna Briggs Institute Center for Evidence-Based Health Care Realistic Evaluation Tool for Case–Control Studies. Overall, the bias risk of the 40 studies included was relatively low. The results of the methodological quality assessment of the included studies are presented in Supplementary Table 4.

### Meta-analysis and descriptive analysis results

**Figure 2:**
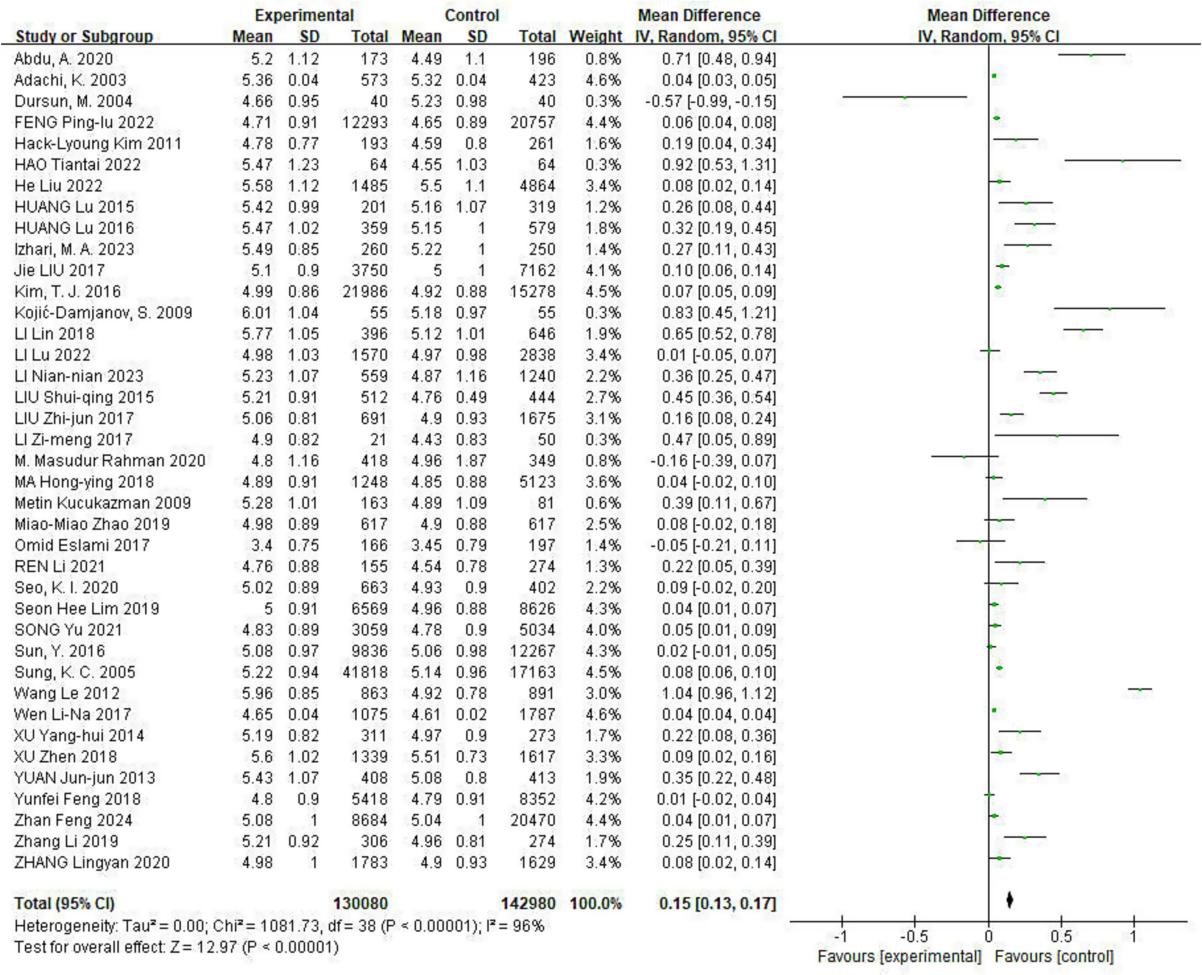
Meta-analysis of Hp infection on abnormally elevated TC levels. Heterogeneity was observed among the studies (*I*^2^ = 96%, *p* < 0.05). TC levels were elevated in Hp-infected patients compared to uninfected patients [TC: MD = 0.15, (95%CI: 0.13, 0.17), *p* < 0.01].

Of the included studies, 39 reported the effect of Hp infection on alterations in TC levels. Heterogeneity was observed among the studies (*I*^2^ = 96%, *p* < 0.05). TC levels were elevated in Hp-infected patients compared to uninfected patients [TC: MD = 0.15, (95%CI: 0.13, 0.17), *p* < 0.01] (Figure 2).

**Figure 3:**
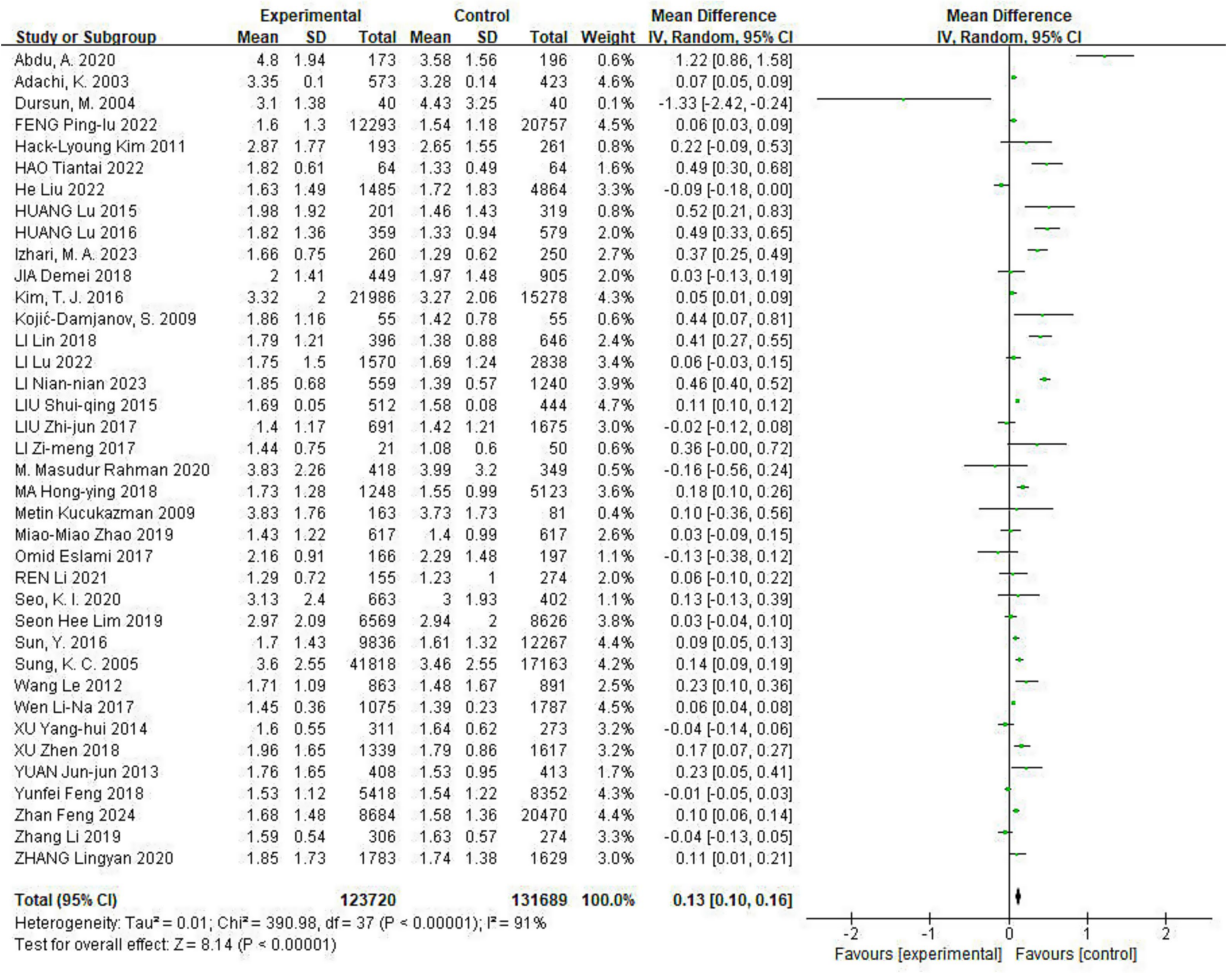
Meta-analysis of Hp infection on abnormally elevated TG levels. Of the included studies, 38 reported the effect of Hp infection on TG levels, with heterogeneity among the studies (*I*^2^ = 91%, *p* < 0.05). TG levels were elevated in Hp-infected patients compared to uninfected patients [TG: MD = 0.13, (95%CI: 0.10, 0.16), *p* < 0.01].

Of the included studies, 38 reported the effect of Hp infection on TG levels, with heterogeneity among the studies (*I*^2^ = 91%, *p* < 0.05). TG levels were elevated in Hp-infected patients compared to uninfected patients [TG: MD = 0.13, (95%CI: 0.10, 0.16), *p* < 0.01] (Figure 3).

**Figure 4:**
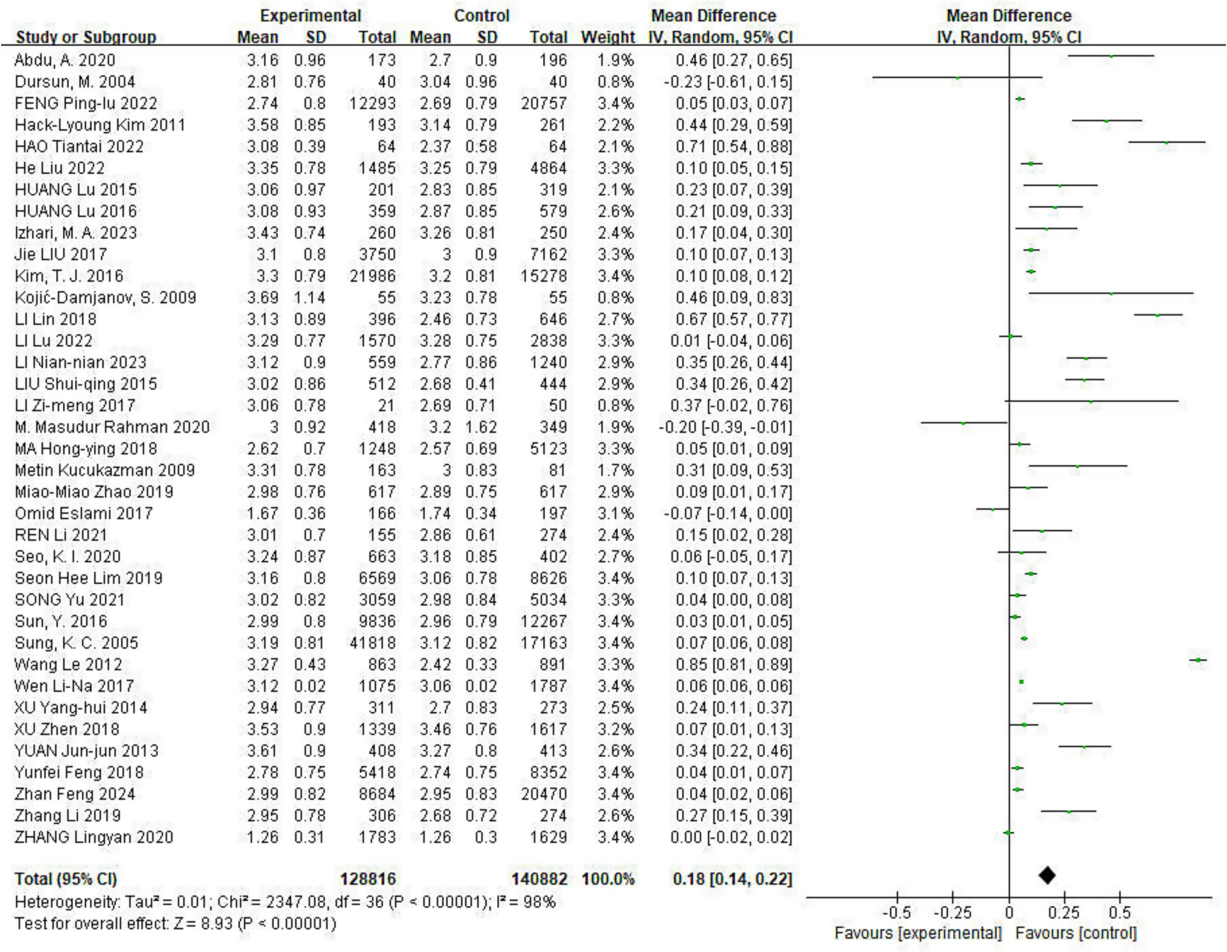
Meta-analysis of Hp infection on abnormally elevated LDL-C levels. Heterogeneity was observed among the studies (*I*^2^ = 98%, *p* < 0.05). LDL-C levels were elevated in Hp-infected patients compared to uninfected patients [LDL-C: MD = 0.18, (95%CI: 0.14, 0.22), *p* < 0.01].

Of the included studies, 37 reported the effect of Hp infection on LDL-C levels. Heterogeneity was observed among the studies (*I*^2^ = 98%, *p* < 0.05). LDL-C levels were elevated in Hp-infected patients compared to uninfected patients [LDL-C: MD = 0.18, (95%CI: 0.14, 0.22), *p* < 0.01] (Figure 4).

**Figure 5:**
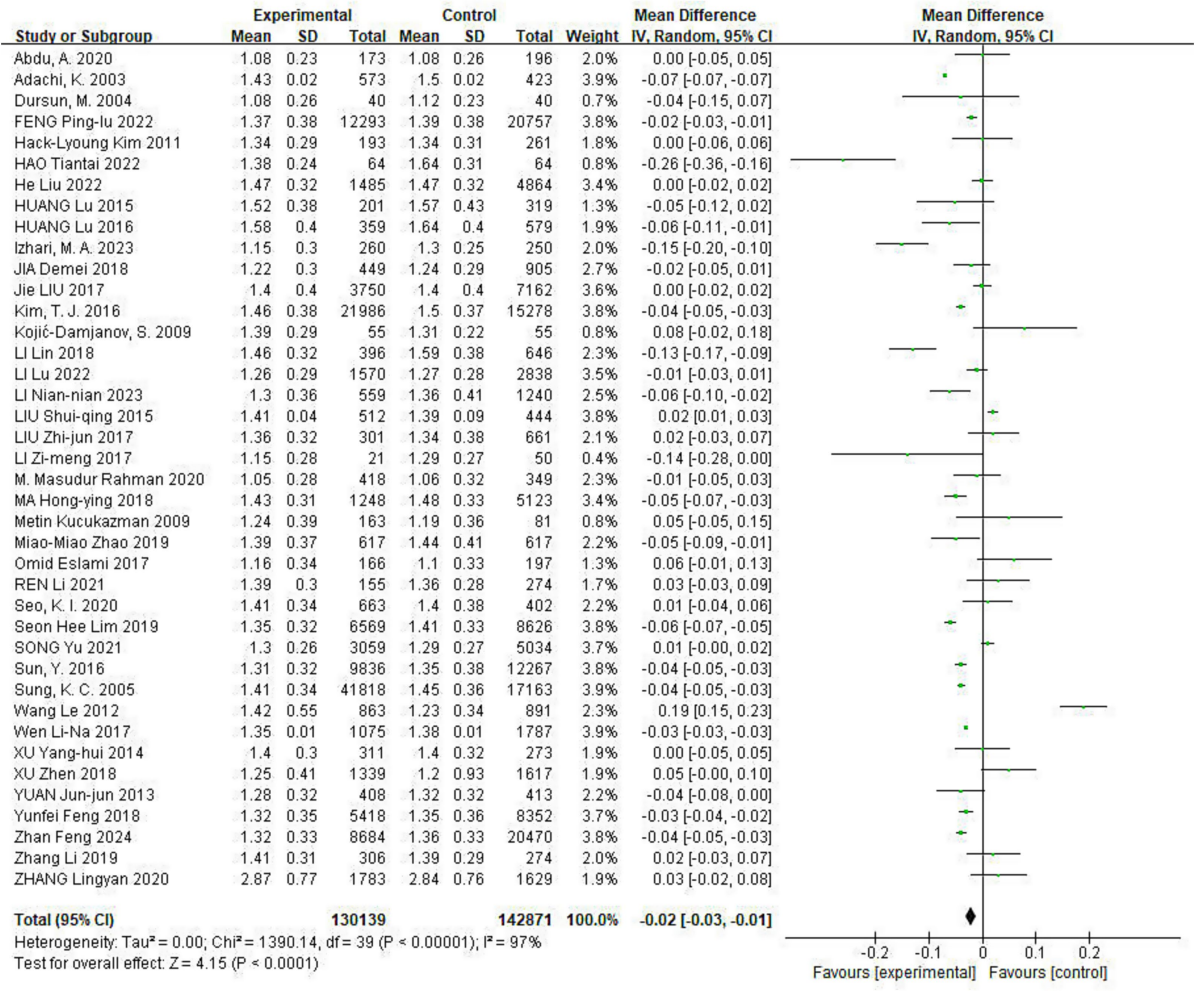
Meta-analysis of Hp infection on abnormally lowered of HDL-C levels. All included studies reported the effect of Hp infection on altered HDL-C levels, heterogeneity existed among different studies (*I*^2^ = 97%, *p* < 0.01). HDL-C levels were lower in Hp-infected patients than in uninfected patients [HDL-C: MD = −0.02, (95%CI: −0.03, −0.01), *p* < 0.05].

All included studies reported the effect of Hp infection on altered HDL-C levels, heterogeneity existed among different studies (*I*^2^ = 97%, *p* < 0.01). HDL-C levels were lower in Hp-infected patients than in uninfected patients [HDL-C: MD = −0.02, (95%CI: −0.03, −0.01), *p* < 0.05] (Figure 5).

### Sensitivity analysis

Sensitivity analyses were conducted using the leave-one-out method to systematically assess the effect of each study on TC, TG, LDL-C, and HDL-C levels. When the study by Adachi (34) was excluded, the TC effect size increased from MD = 0.15 (95%CI: 0.13–0.17) to MD = 0.18 (95%CI: 0.14–0.21), with a 95% confidence interval that still did not include the null value of 0, suggesting that the effect size remained statistically significant. A deeper exploration revealed that the relatively small sample size of the study and the significantly higher baseline level of TC in the study population than in the other included studies may have contributed to the heterogeneity. Notably, the direction of effect sizes and the statistical significance of TG, HDL-C, and LDL-C levels did not show substantial changes after the sequential exclusion of the remaining studies. This suggests that the results of the analyses regarding TG, LDL-C, and HDL-C in this study were robust, whereas, for the TC indices, the statistical significance of the overall effect sizes remained, although fluctuations in effect sizes were likely due to a single study.

### Subgroup analysis and meta-regression

The results of the subgroup analysis showed that the effect of Hp infection on the abnormal decrease in HDL-C levels in non-Asian countries was more significant than in Asian countries. However, the effects of Hp infection on the abnormal elevation of TC, TG, and LDL-C levels did not differ significantly between Asian and non-Asian countries (Table 1).

**Table 1:** Sub-group analysis of different regions.

Variate	Number of studies	MD, 95% CI	Heterogeneity test	
			*I*^2^ (%)	*p*
TC				
Asian countries	37	0.14 (0.12, 0.17)	97	<0.05
Non-Asian countries	2	0.74 (0.55, 0.94)	0	<0.05
Total	39	0.15 (0.13, 0.17)	97.2	<0.05
TG				
Asian countries	36	0.12 (0.09, 0.15)	90	<0.05
Non-Asian countries	2	0.83 (0.07, 1.06)	89	<0.05
Total	38	0.13 (0.10, 0.16)	70.1	>0.05
LDL-C				
Asian countries	35	0.17 (0.13, 0.21)	99	<0.05
Non-Asian countries	2	0.46 (0.29, 0.63)	0	<0.05
Total	37	0.18 (0.14, 0.22)	90.8	<0.05
HDL-C				
Asian countries	38	−0.02 (−0.03, −0.01)	97	<0.05
Non-Asian countries	2	0.03 (−0.05, 0.01)	52	>0.05
Total	40	−0.02 (−0.03, −0.01)	42.7	>0.05

Subgroup analysis of different countries showed that Hp infection significantly increased TC, TG, and LDL-C levels in Ethiopia, Serbia, China, Saudi Arabia, and Turkey. It also showed that Hp infection significantly decreased HDL-C levels in Japan, Korea, China, and Saudi Arabia compared to other countries (Supplementary Table 5).

Subgroup analyses of different research types showed that both cross-sectional and case–control studies reported increased levels of TC, TG, and LDL-C and decreased HDL-C levels (Supplementary Table 6).

To detect the sources of heterogeneity, a random-effects meta-regression was run using sex ratio, average age, and BMI. The data included in the sex ratio were the proportions of males. The analysis indicated a negative correlation between the sex ratio and the TC indicator. As the number of men increased, TC indicator values tended to decrease. Furthermore, BMI and HDL-C indicators were negatively correlated; the higher the BMI value, the more the value of HDL-C indicators tended to decrease. No significant correlation was found between average age and dyslipidemia (Table 2).

**Table 2:** Meta regression of Ratios of sex, Average age and BMI.

Variate	TC			TG			LDL-C			HDL-C		
	SE	95%CI	*p*	SE	95%CI	*p*	SE	95%CI	*p*	SE	95%CI	*p*
Ratios of sex	0.05	(−0.38, −0.17)	<0.05	0.39	(−0.98, 0.53)	>0.05	0.34	(−0.81, 0.54)	>0.05	0.04	(−0.12, 0.01)	>0.05
Average age	0.00	(−0.01, −0.00)	<0.05	0.00	(−0.01, −0.00)	<0.05	0.00	(−0.00, 0.00)	>0.05	0.00	(−0.01, −0.00)	<0.05
BMI	0.08	(−0.10, 0.23)	>0.05	0.05	(−0.05, 0.16)	>0.05	0.06	(−0.04, 0.21)	>0.05	0.02	(−0.08, −0.01)	<0.05

Ratios of sex included data for males.

### Publication bias

Funnel plots were used to test for publication bias. The funnel plots in this study illustrated a symmetrical distribution, suggesting no obvious publication bias (Supplementary Figures 1–4).

Begg’s and Egger’s tests were used to test for publication bias (TC Begg’s test, *p* = 0.068; Egger’s test, *p* = 0.001; TG: Begg’s test, *p* = 0.085; Egger’s test, *p* = 0.346; LDL-C: Begg’s test, *p* = 0.071; Egger’s test, *p* = 0.042; and HDL-C: Begg’s test, *p* = 0.852; Egger’s test, *p* = 0.709). Begg’s test results for both TC and LDL-C levels were *p* < 0.05, suggesting a publication bias. For TC and LDL-C, after deleting the related studies of Adachi (34) and Wang and Zhao (58), the potential publication bias was further corrected by the trim and fill method. The funnel plots were symmetrical after trimming (Supplementary Figures 5, 6), suggesting that the publication bias of the related studies of Adachi (34) and Wang and Zhao (58) had less impact on the results of the present study and that the results were relatively robust. The results of Egger’s and Begg’s tests for TG and HDL-C both showed *p* > 0.05, indicating no significant publication bias.

## Discussion

This study has represented the systematic review and meta-analysis to analyze the relationship between Hp infection and dyslipidemia in populations stratified by Asian and outside Asia regions. The results in this study have indicated that Hp infection was correlated with abnormally elevated levels of TC, TG, and LDL-C and abnormally lowered HDL-C levels. Another meta-analysis that included 27 studies from around the world reported similar results, with a significant association between Hp infection and changes in serum lipid profile (63). A recent meta-analysis showed that Hp infection was associated with increased TC and LDL levels and decreased HDL levels; however, the analysis failed to demonstrate a statistically significant association between Hp infection and TG levels. This discrepancy may be due to the exclusion of non-English publications, which could introduce publication bias and overlook publications that could have potentially limited the comprehensiveness of the analysis, since studies conducted in non-English-speaking regions may not have been included (64). The current study was rigorously screened, and the quality of the included literature was high overall, covering research from different regions, enhancing representativeness and credibility and providing a more realistic picture of the status of the relationship between Hp infection and dyslipidemia worldwide.

This study found that Hp infection promotes dyslipidemia by increasing TC, TG, and LDL-C levels. Several studies reported similar results (32, 36, 56). After Hp colonization of the gastric mucosa, Hp lipopolysaccharide stimulates the production of inflammatory cytokines such as TNF-α, IL-1, IL-6 and so on (65, 66). Subsequently, the upregulation of inflammatory cytokines induced by Hp infection impairs lipid metabolism, and this chronic inflammatory process may indirectly lead to endothelial cell damage, thereby promoting atherosclerosis development (67). Lipoprotein lipase activity is inhibited by TNF-α, causing lipids to shift out of tissues, raising blood TC levels, and promoting the secretion of very low-density lipoproteins and the apolipoproteins of LDL. Consequently, TG and LDL-C concentrations increase, leading to dyslipidemia (68). Although TC, TG, and LDL-C levels in Hp-infected patients show only mildly elevated abnormalities, even slight changes in blood lipids are significantly associated with the risk of cardiovascular diseases such as myocardial infarction (69). Specifically, for every 1 mmol/L increase in TC levels above the “normal” range, the risk of coronary heart disease has increased by 41% and the risk of ischemic stroke increases by 23% (70). Moreover, for every 1 mmol/L increase in TG and LDL-C levels, the risk of aortic valve stenosis has increased by 38 and 52%, respectively (71).

This study demonstrated a small but statistically significant decrease in HDL levels in Hp-infected individuals. Similar studies have reported consistent results (34). HDL cholesterol is now considered one of the major protective mechanisms against atherosclerosis, which promotes the net movement of cholesterol from peripheral tissues back to the liver via the reverse cholesterol transport pathway. HDL-C plays a crucial anti-inflammatory role, regulating the expression and release of inflammatory factors and reducing ox-LDL damage to vascular endothelial cells and antithrombotic agents (72, 73). Given that HDL is protective against cardiovascular diseases by facilitating reverse cholesterol transport, even a marginal reduction in HDL levels due to Hp infection could contribute to an increased cardiovascular risk in affected individuals (74). Hp infection may lead to reduced HDL-C levels through several mechanisms. Hp infection may promote the secretion of gamma interferon and thrombin, which trigger a chronic inflammatory response in the organism. Furthermore, TNF-α inhibits the activity of lipoprotein esterase, which in turn reduces the synthesis of HDL-C (75, 76). Similarly, Hp infection may also prompt hepatic secretion of acute chronotropic proteins, such as c-reactive protein, which accelerates the metabolic process of HDL-C, further reducing HDL-C levels (77, 78). HDL levels are also sensitive to lifestyle and dietary habits; thus, future studies should consider adjusting for these variables to assess the independent impact of Hp on HDL more accurately.

The association between Hp infection and dyslipidemia may be mediated through complex multifactorial mechanisms. Chronic inflammation such as Hp infection releases high levels of pro-inflammatory cytokines such as IL-6 and TNF-α, which may increase hepatic lipid synthesis and impair lipid clearance, thereby increasing cholesterol and LDL levels while potentially decreasing HDL concentrations (79). Additionally, specific Hp strains express virulence factors such as CagA, which elicit a more intense inflammatory response and are associated with severe metabolic disturbances, including altered lipid metabolism (80). Hp infection is also associated with insulin resistance, which is a precursor of metabolic syndrome and an independent risk factor for dyslipidemia (81). Insulin resistance may exacerbate dyslipidemia through mechanisms such as increased hepatic triglyceride synthesis and impaired lipid oxidation (82). A large body of evidence suggests that Hp infection can alter the composition of intestinal flora, leading to changes that may affect lipid metabolism (83, 84).

Hp infection has a more significant effect on the abnormal reduction in HDL-C levels in non-Asian than in Asian countries. This disparity is likely influenced by multiple factors, with diet being a prominent contributor. Non-Asian populations commonly consume diets rich in saturated and trans-fats, including foods such as butter, cheese, and fried items. Excessive intake of these substances significantly contributes to a decrease in HDL-C levels since they disrupt the lipid metabolism pathways (85). In contrast, traditional Asian diets predominantly contain grains, vegetables, and fish, with a relatively lower fat content. This dietary pattern promotes an increase in HDL-C levels, helping mitigate the negative effects of Hp infection on lipid profiles (86). Furthermore, potential contributions from genetic factors warrant consideration. Genetic polymorphisms in cholesteryl ester transfer protein (CETP) may explain observed regional variations in HDL-C levels. Genetic defects in CETP are more prevalent in East Asian populations, primarily manifested as D442G mutant and Int14A mutant abnormalities, which correspond to significantly reduced CETP activity and elevated HDL-C levels (87). Socioeconomic disparities also may influence the measurement results and interpretation of HDL-C levels in Hp-infected individuals across different regions. In non-Asian regions, despite lower Hp infection rates, robust healthcare systems enable earlier detection of mild HDL-C abnormalities in infected individuals (88).

This study had several limitations. Some of the included studies did not adequately control for confounding factors such as dietary habits, physical activity, and genetic background and were not comprehensively considered. Despite the use of methods such as subgroup analysis to explore the sources of heterogeneity, the heterogeneity in this study could not be fully explained or eliminated. Due to the small sample size of non-Asian countries included in this study, more studies in different regions are needed to investigate the mechanism of Hp infection in HDL-C reduction.

## Conclusion

This study confirmed that Hp infection was correlated with abnormally elevated TC, TG, and LDL-C levels and abnormally lowered HDL-C levels. The effect of Hp infection on dyslipidemia varies in different regions, such as Asia.

## Data Availability

The original contributions presented in the study are included in the article/supplementary material, further inquiries can be directed to the corresponding author.

## Supplementary material

Supplementary FIGURE 1 Funnel plot of elevated TC levels due to Hp infection.

Supplementary FIGURE 2 Funnel plot of elevated TG levels due to Hp infection.

Supplementary FIGURE 3 Funnel plot of elevated LDL-C levels due to Hp infection.

Supplementary FIGURE 4 Funnel plot of reduced HDL-C levels due to Hp infection.

Supplementary FIGURE 5 Funnel plot of TC after using trim and fill method.

Supplementary FIGURE 6 Funnel plot of LDL-C after using trim and fill method.
