# Supplementary figures and images for: The effect of Hp infection on dyslipidemia in Asia and out of Asia: a systematic review and meta-analysis
Source: Front Med (Lausanne). 2025 Oct 16;12:1643218. doi: 10.3389/fmed.2025.1643218 (PMC12571730; doi:10.3389/fmed.2025.1643218)

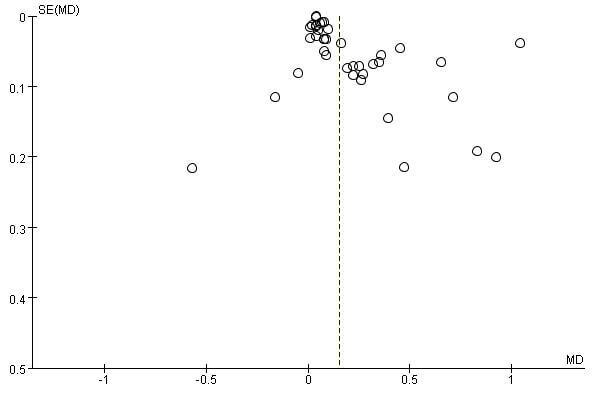

Supplement: Supplementary FIGURE 1 — Funnel plot of elevated TC levels due to Hp infection. [file Image_1.jpeg]

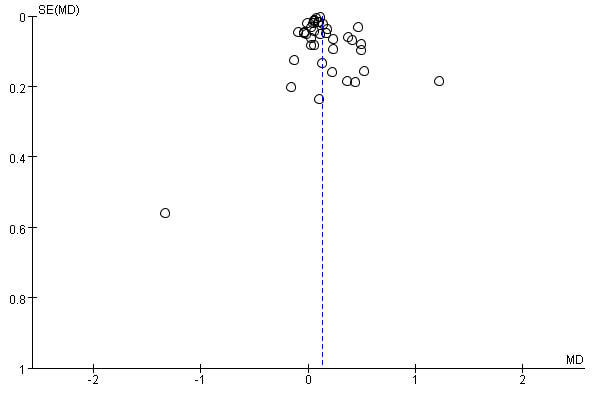

Supplement: Supplementary FIGURE 2 — Funnel plot of elevated TG levels due to Hp infection. [file Image_2.jpeg]

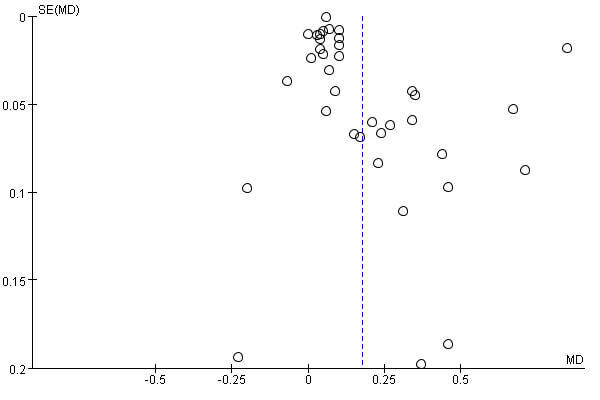

Supplement: Supplementary FIGURE 3 — Funnel plot of elevated LDL-C levels due to Hp infection. [file Image_3.jpeg]

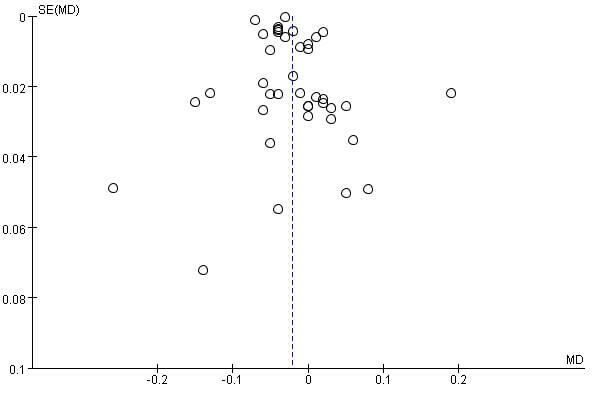

Supplement: Supplementary FIGURE 4 — Funnel plot of reduced HDL-C levels due to Hp infection. [file Image_4.jpeg]

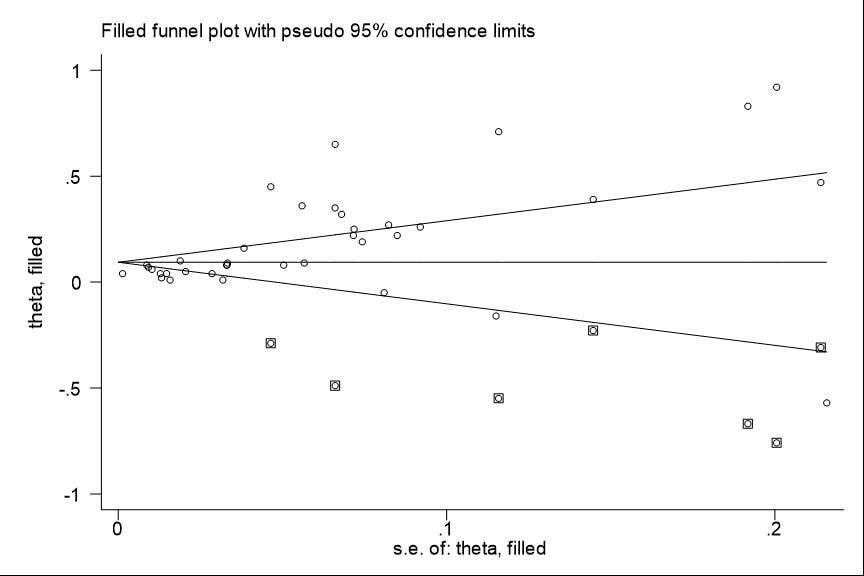

Supplement: Supplementary FIGURE 5 — Funnel plot of TC after using trim and fill method. [file Image_5.jpeg]

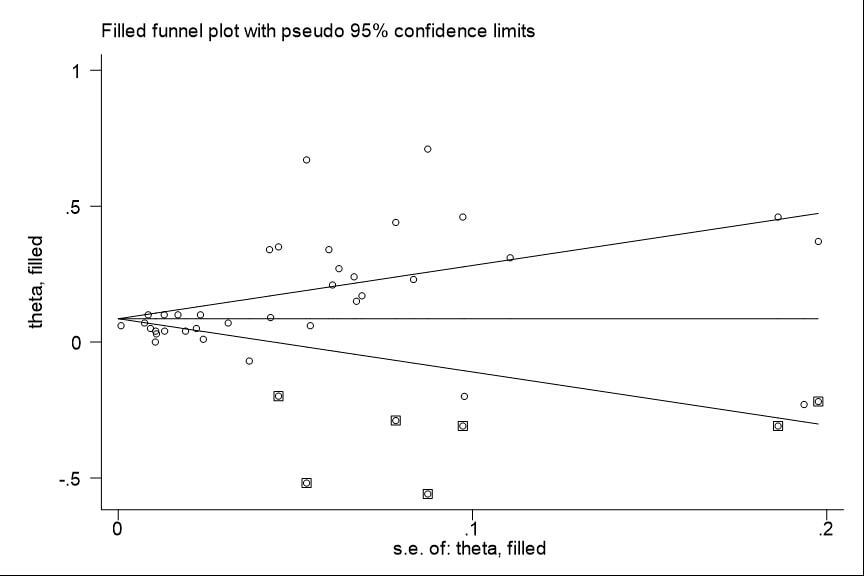

Supplement: Supplementary FIGURE 6 — Funnel plot of LDL-C after using trim and fill method. [file Image_6.jpeg]
