# Supplementary material for: The effect of Hp infection on dyslipidemia in Asia and out of Asia: a systematic review and meta-analysis
Source: Front Med (Lausanne). 2025 Oct 16;12:1643218. doi: 10.3389/fmed.2025.1643218 (PMC12571730; doi:10.3389/fmed.2025.1643218)
Supplement: Supplementary file 7 [file Data_Sheet_1.docx]

**Supplementary tables**

**Supplementary table 1 Search strategies in this study**

|  | **Search Strategies** |
| --- | --- |
| #1 | (Helicobacter pylori[MeSH Terms]) OR (Helicobacter Infections[MeSH Terms]) |
| #2 | ((((((((Helicobacter pylori) OR (Helicobacter Infections)) OR (Helicobacter nemestrinae)) OR (Campylobacter pylori)) OR (Campylobacter pylori subsp. Pylori)) OR (Campylobacter pyloridis)) OR (Infections, Helicobacter)) OR (Helicobacter Infection)) OR (Infection, Helicobacter) |
| #3 | #1 OR #2 |
| #4 | Dyslipidemias[MeSH Terms] |
| #5 | (((((((Dyslipidemias) OR (Dyslipidemia)) OR (Dyslipoproteinemias)) OR (Dyslipoproteinemia)) OR (High total cholesterol)) OR (Hypertriglyceridemia)) OR (decrease of HDL cholesterol)) OR (Elevated LDL cholesterol) |
| #6 | #4 OR #5 |
| #7 | #3 AND #6 |

**Supplementary table 2 Inclusion and exclusion criteria**

|  | **Inclusion criteria** |  | **Exclusion criteria** |
| --- | --- | --- | --- |
| **P** | Adults over 18 years of age diagnosed with Hp infection. No restrictions were applied regarding sex or ethnicity |  | Individuals receiving anti-Hp therapy, lipid-lowering therapy, or antibiotic therapy were excluded |
| **E** | Hp infection status served as the primary exposure factor |  | Participants with comorbidities known to affect lipid metabolism—namely, coronary heart disease, diabetes mellitus, metabolic syndrome, severe liver or kidney disease, or malignant tumors |
| **C** | Healthy control groups comprised individuals without Hp infection, matched by age and sex |  | Under 18 years of age, non suffering from Hp infected |
| **O** | Indicators included plasma levels of TC, TG, LDL-C, and HDL-C, to compare these levels between Hp-infected participants and non-infected controls |  | Studies lacking essential data or for which such data were unavailable were excluded |
| **S** | All types of primary research exploring the association between Hp infection and dyslipidemia were included, specifically cross-sectional studies, case-control studies, and cohort studies |  | Literature that could not be retrieved in full, along with secondary research types (e.g., reviews, meta-analyses), conference abstracts, academic reports, guidelines, protocols, animal studies, and cellular experiments |

**Supplementary table 3 The characteristics of the included studies (n=40)**

| First Author | Year | Country | Research Type | Sample Size（Total/ Hp^+^/ Hp^-^) | Hp Detection Method | Lipid levels in Hp-positive (mmol/L,‾x±s) | | | | Lipid levels in Hp-negative (mmol/L,‾x±s) | | | | Findings |
| --- | --- | --- | --- | --- | --- | --- | --- | --- | --- | --- | --- | --- | --- | --- |
|  |  |  |  |  |  | TC | TG | LDL-C | HDL-C | TC | TG | LDL-C | HDL-C |  |
| Seo KI ^[18]^ | 2020 | Korea | Case-control study | 1065/663/402 | The rapid urease | 5.02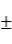0.89 | 3.13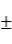2.40 | 3.24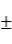0.87 | 1.41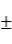0.34 | 4.93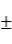0.90 | 3.00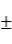1.93 | 3.18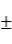0.85 | 1.4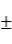0.38 | Hp infection could affect lipid profiles and may be different by sex. |
| Abdu A ^[19]^ | 2020 | Ethiopia | Cross-sectional study | 369/173/196 | Antibody dipstick for serum/plasma | 5.20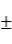1.12 | 4.80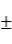1.94 | 3.16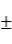0.96 | 1.08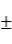0.23 | 4.49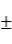1.10 | 3.58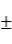1.56 | 2.70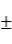0.90 | 1.08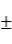0.26 | Hp infection may have an effect on dyslipidemia changes. |
| Izhari MA ^[20]^ | 2023 | Saudi Arabia | Case-control study | 510/260/250 | Stool antigen test | 5.49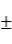0.85 | 1.66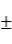0.75 | 3.43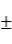0.74 | 1.15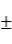0.30 | 5.22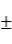1.00 | 1.29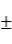0.62 | 3.26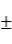0.81 | 1.3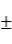0.25 | Hp infection increases the risk of dyslipidemia. |
| Kim TJ ^[21]^ | 2016 | Korea | Cross-sectional study | 37264/21986/15278 | Serological test | 4.99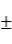0.86 | 3.32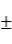2.00 | 3.30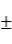0.79 | 1.46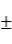0.38 | 4.92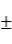0.88 | 3.27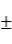2.06 | 3.20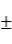0.81 | 1.50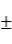0.37 | Hp infection has an effect on dyslipidemia. |
| Huang L ^[27]^ | 2015 | China | Cross-sectional study | 520/201/319 | ^13^C-UBT | 5.42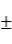0.99 | 1.98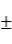1.92 | 3.06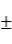0.97 | 1.52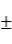0.38 | 5.16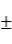1.07 | 1.46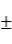1.43 | 2.83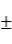0.85 | 1.57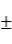0.43 | Hp infection has an effect on dyslipidemia. |
| Li NN ^[28]^ | 2023 | China | Case-control study | 1799/559/1240 | ^14^C-UBT | 5.23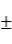1.07 | 1.85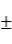0.68 | 3.12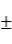0.90 | 1.3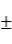0.36 | 4.87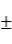1.16 | 1.39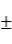0.57 | 2.77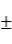0.86 | 1.36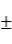0.41 | Hp infection is closely related to dyslipidemia. |
| Wen LN ^[29]^ | 2017 | China | Case-control study | 2862/1075/1787 | ^13^C-UBT | 4.65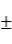0.04 | 1.45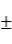0.36 | 3.12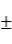0.02 | 1.35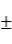0.01 | 4.61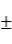0.02 | 1.39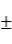0.23 | 3.06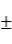0.02 | 1.38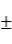0.01 | Hp infection has an effect on dyslipidemia. |
| Xu Z ^[30]^ | 2018 | China | Case-control study | 2956/1339/1617 | ^14^C-UBT | 5.60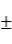1.02 | 1.96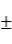1.65 | 3.53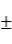0.90 | 1.25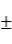0.41 | 5.51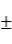0.73 | 1.79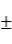0.86 | 3.46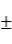0.76 | 1.20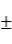0.93 | Hp infection is associated with dyslipidemia. |
| Yuan JJ ^[31]^ | 2013 | China | Cross-sectional study | 821/408/413 | Serological test | 5.43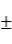1.07 | 1.76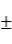1.65 | 3.61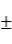0.90 | 1.28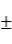0.32 | 5.08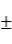0.80 | 1.53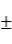0.95 | 3.27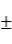0.80 | 1.32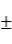0.32 | Hp infection has an effect on dyslipidemia. |
| Jia DM ^[32]^ | 2018 | China | Cross-sectional study | 1354/449/905 | ^13^C-UBT | No data | 2.00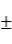1.41 | No data | 1.22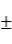0.30 | No data | 1.97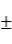1.48 | No data | 1.24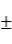0.29 | Hp infection affects the incidence of abnormal TC elevation. |
| Zhang LY ^[33]^ | 2020 | China | Case-control study | 3412/1783/1629 | ^14^C-UBT | 4.98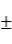1.00 | 1.85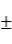1.73 | 1.26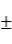0.31 | 2.87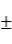0.77 | 4.90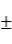0.93 | 1.74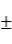1.38 | 1.26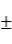0.30 | 2.84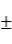0.76 | There was no significant effect between Hp infection and dyslipidemia. |
| Adachi K ^[34]^ | 2003 | Japan | Cross-sectional study | 996/573/423 | Serological test | 5.36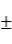0.04 | 3.35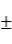0.10 | No data | 1.43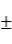0.02 | 5.32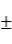0.04 | 3.28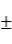0.14 | No data | 1.50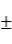0.02 | The serum HDLC level was lower in Hp-seropositive than seronegative Japanese individuals. |
| Dursun M ^[35]^ | 2004 | Turkey | Cross-sectional study | 80/40/40 | Endoscopic gastric mucosal biopsies | 4.66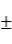0.95 | 3.10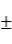1.38 | 2.81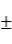0.76 | 1.08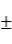0.26 | 5.23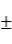0.98 | 4.43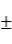3.25 | 3.04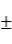0.96 | 1.12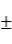0.23 | Hp infection seems to be metabolically neutral in terms of serum lipids (TC, LDL-C, HDL-C and TG) in non-obese young adult males. |
| Feng PL ^[36]^ | 2022 | China | Cross-sectional study | 33050/12293/20757 | ^13^C-UBT,^14^C-UBT | 4.71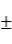0.9 | 1.6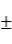1.3 | 2.740.8 | 1.370.38 | 4.650.89 | 1.541.18 | 2.690.79 | 1.390.38 | Hp infection is a risk factor for low HDL-C and high LDL-C. |
| Kim HL ^[37]^ | 2011 | Korea | Cross-sectional study | 454/193/261 | Endoscopic gastric mucosal biopsies | 4.780.77 | 2.871.77 | 3.580.85 | 1.340.29 | 4.590.8 | 2.651.55 | 3.140.79 | 1.340.31 | Hp infection is associated with the elevated serum LDL-C levels in elderly Koreans. |
| Hao TT ^[38]^ | 2022 | China | Case-control study | 128/64/64 | ^14^C-UBT | 5.471.23 | 1.820.61 | 3.080.39 | 1.380.24 | 4.551.03 | 1.330.49 | 2.370.58 | 1.640.31 | Hp infection may lead to dyslipidemia. |
| Liu H ^[39]^ | 2022 | China | Cross-sectional study | 6349/1485/4864 | ^14^C-UBT | 5.581.12 | 1.631.49 | 3.350.78 | 1.470.32 | 5.51.10 | 1.721.83 | 3.250.79 | 1.470.32 | Hp infection affected the lipids’ levels. |
| Huang L ^[40]^ | 2016 | China | Cross-sectional study | 938/359/579 | ^13^C-UBT | 5.471.02 | 1.821.36 | 3.080.93 | 1.580.40 | 5.151.00 | 1.330.94 | 2.870.85 | 1.640.40 | Hp infection is associated with dyslipidemia. |
| Liu J ^[41]^ | 2017 | China | Cross-sectional study | 10912/3750/7162 | ^13^C-UBT | 5.100.90 | No data | 3.100.80 | 1.400.40 | 5.001.00 | No data | 3.000.90 | 1.400.40 | Hp infection affects TC, LDL-C level. |
| Kojić-Damjanov S ^[42]^ | 2009 | Serbia | Cross-sectional study | 110/55/55 | ELISA | 6.011.04 | 1.861.16 | 3.691.14 | 1.390.29 | 5.180.97 | 1.420.78 | 3.230.78 | 1.310.22 | Hp infection could modify the lipid profile in a proatherogenic way. |
| Li L ^[43]^ | 2018 | China | Cross-sectional study | 1042/396/646 | Serological test | 5.771.05 | 1.791.21 | 3.130.89 | 1.460.32 | 5.121.01 | 1.380.88 | 2.460.73 | 1.590.38 | Dyslipidemia is associated with Hp infection. |
| Li L ^[44]^ | 2022 | China | Cross-sectional study | 4408/1570/2838 | ^13^C-UBT | 4.981.03 | 1.751.50 | 3.290.77 | 1.260.29 | 4.970.98 | 1.691.24 | 3.280.75 | 1.270.28 | Hp infection may have an effect on dyslipidemia changes. |
| Li ZM ^[45]^ | 2017 | China | Cross-sectional study | 71/21/50 | ^14^C-UBT | 4.900.82 | 1.440.75 | 3.060.78 | 1.150.28 | 4.430.83 | 1.080.60 | 2.690.71 | 1.290.27 | Hp infection linked to dyslipidemia. |
| Liu SQ ^[46]^ | 2015 | China | Cross-sectional study | 956/512/444 | ^14^C-UBT | 5.210.91 | 1.690.05 | 3.020.86 | 1.410.04 | 4.760.49 | 1.580.08 | 2.680.41 | 1.390.09 | Hp infection causes abnormal lipid metabolism. |
| Liu ZJ ^[47]^ | 2017 | China | Cross-sectional study | 2366/691/1675 | ^14^C-UBT | 5.060.81 | 1.41.17 | No data | 1.360.32 | 4.900.93 | 1.421.21 | No data | 1.340.38 | The relationship between Hp infection and dyslipidemia is unclear. |
| Rahman MM ^[48]^ | 2020 | Bangladesh | Cross-sectional study | 767/418/349 | Serological test | 4.801.16 | 3.832.26 | 3.000.92 | 1.050.28 | 4.961.87 | 3.993.20 | 3.201.62 | 1.060.32 | Hp seropositive subjects had significantly lower serum LDL-C compared to seronegative subjects. |
| Ma HY ^[49]^ | 2018 | China | Case-control study | 6371/1248/5123 | Serological test | 4.890.91 | 1.731.28 | 2.620.70 | 1.430.31 | 4.850.88 | 1.550.99 | 2.570.69 | 1.480.33 | Hp infection is strongly associated with lipid metabolism. |
| Kucukazman M ^[50]^ | 2009 | Turkey | Cross-sectional study | 244/163/81 | Endoscopic gastric mucosal biopsies | 5.281.01 | 3.831.76 | 3.310.78 | 1.240.39 | 4.891.09 | 3.731.73 | 3.000.83 | 1.190.36 | Increased levels of TC and LDL-C were found in patients infected with Hp. |
| Zhao MM ^[51]^ | 2019 | China | Cross-sectional study | 1234/617/617 | ^13^C-UBT | 4.980.89 | 1.431.22 | 2.980.76 | 1.390.37 | 4.90.88 | 1.40.99 | 2.890.75 | 1.440.41 | Hp infection is an independent risk factor for less favorable lipid profiles. |
| Eslami O ^[52]^ | 2017 | Iran | Cross-sectional study | 363/166/197 | ELISA | 3.40.75 | 2.160.91 | 1.670.36 | 1.160.34 | 3.450.79 | 2.291.48 | 1.740.34 | 1.100.33 | Hp infection is no significant difference was found in mean levels of TC, TG and HDL‑C. |
| Ren L ^[53]^ | 2021 | China | Cross-sectional study | 429/155/274 | ^14^C-UBT | 4.760.88 | 1.290.72 | 3.010.70 | 1.390.30 | 4.540.78 | 1.231.00 | 2.860.61 | 1.360.28 | Hp infection is associated with elevated TC and LDL-C. |
| Lim SH ^[54]^ | 2019 | Korea | Cross-sectional study | 15195/6569/8626 | Serological test | 5.000.91 | 2.972.09 | 3.160.80 | 1.350.32 | 4.960.88 | 2.942.00 | 3.060.78 | 1.410.33 | TC, LDL-C, and HDL-C were significantly different between subjects with and without Hp seropositivity and were significantly associated with HP infection. |
| Song Y ^[55]^ | 2021 | China | Case-control study | 8093/3059/5034 | ^13^C-UBT | 4.830.89 | No data | 3.200.82 | 1.300.26 | 4.780.90 | No data | 2.980.84 | 1.290.27 | High TC and obesity are risk factors for Hp infection. |
| Sun Y ^[56]^ | 2016 | China | Case-control study | 22103/9836/12267 | ^13^C-UBT | 5.080.97 | 1.701.43 | 2.990.80 | 1.310.32 | 5.060.98 | 1.611.32 | 2.960.79 | 1.350.38 | The relationship between HP infection and abnormal TG and HDL-C was the most significant. There was no significant difference with TC and LDL-C. |
| Sung KC ^[57]^ | 2005 | Korea | Cross-sectional study | 58981/41818/17163 | ELISA | 5.220.94 | 3.602.55 | 3.190.81 | 1.410.34 | 5.140.96 | 3.462.55 | 3.120.82 | 1.450.36 | Hp infection is associated with cardiovascular risk factors, especially with TC, HDL-C and apolipoproteins. |
| Wang L ^[58]^ | 2012 | China | Case-control study | 1754/863/891 | Serological test | 5.960.85 | 1.711.09 | 3.270.43 | 1.420.55 | 4.920.78 | 1.481.67 | 2.420.33 | 1.230.34 | Hp infection was significantly associated with elevated LDL-C levels. |
| Xu YH ^[59]^ | 2014 | China | Cross-sectional study | 584/311/273 | ^13^C-UBT、^14^C-UBT | 5.190.82 | 1.600.55 | 2.940.77 | 1.400.30 | 4.970.90 | 1.640.62 | 2.700.83 | 1.400.32 | Hp infection may be linked to dyslipidemia. |
| Feng Y ^[60]^ | 2018 | China | Cross-sectional study | 13770/5418/8352 | ^13^C-UBT | 4.800.90 | 1.531.12 | 2.780.75 | 1.320.35 | 4.790.91 | 1.541.12 | 2.740.75 | 1.350.36 | Hp infection affected the LDL-C, HDL-C levels. |
| Feng Z ^[61]^ | 2024 | China | Case-control study | 29154/8684/20470 | ^13^C-UBT | 5.081.00 | 1.681.48 | 2.990.82 | 1.320.33 | 5.041.00 | 1.581.36 | 2.950.83 | 1.360.33 | Hp infection might has a significant impact on metabolic abnormalities. |
| Zhang L ^[62]^ | 2019 | China | Cross-sectional study | 580/306/274 | ^13^C-UBT、^14^C-UBT | 5.210.92 | 1.590.54 | 2.950.78 | 1.410.31 | 4.960.81 | 1.630.57 | 2.680.72 | 1.390.29 | Hp infection has little to do with dyslipidemia, while Hp infection is associated with TC and LDL-C. |

**Note:** Hp, Helicobacter pylori; TC, Total cholesterol; TG, triglycerides; LDL-C, low-density lipoprotein cholesterol; HDL-C, high-density lipoprotein cholesterol; ‾x, mean; s, standard deviation; ELISA, enzyme-linked immunosorbent assay; ^13^C-UBT, Carbon 13 Urea Breath Test; ^14^C-UBT, Carbon 14 Urea Breath Test.

**Supplementary Table 4 The results of the methodological quality assessment of the included studies (n=40)**

| **First Author** | **comparability of cases and controls apart from exposure** | **appropriate matching between cases and controls** | **use of the same criteria for the recruitment of cases and controls** | **use of standard, valid, and reliable methods to measure exposure** | **use of the same methods to measure exposure in both cases and controls** | **consideration of confounding factors** | **control of confounding factors** | **use of standard, valid, and reliable methods to measure outcomes** | **adequacy of the exposure duration** | **appropriate statistical methods for data analysis** |
| --- | --- | --- | --- | --- | --- | --- | --- | --- | --- | --- |
| Abdu A | N/C | Yes | Yes | Yes | Yes | Yes | Yes | Yes | N/A | Yes |
| Adachi K | Yes | Yes | Yes | Yes | Yes | Yes | Yes | N/C | N/A | Yes |
| Dursun M | Yes | Yes | Yes | Yes | Yes | No | No | N/C | N/A | Yes |
| Feng PL | N/C | Yes | Yes | Yes | Yes | Yes | Yes | Yes | N/A | Yes |
| Kim HL | Yes | Yes | Yes | Yes | Yes | Yes | Yes | Yes | N/A | Yes |
| Hao TT | Yes | Yes | Yes | Yes | Yes | No | No | Yes | N/A | Yes |
| Liu H | Yes | Yes | Yes | Yes | Yes | Yes | Yes | Yes | N/A | Yes |
| Huang L (2015) | Yes | Yes | Yes | Yes | Yes | No | No | Yes | N/A | Yes |
| Huang L (2016) | Yes | Yes | Yes | Yes | Yes | Yes | Yes | Yes | N/A | Yes |
| Izhari MA | Yes | Yes | Yes | Yes | Yes | Yes | Yes | Yes | N/A | Yes |
| Jia DM | Yes | Yes | Yes | Yes | Yes | Yes | Yes | Yes | N/A | Yes |
| Liu J | Yes | Yes | Yes | Yes | Yes | Yes | Yes | Yes | N/A | Yes |
| Kim TJ | N/C | Yes | Yes | Yes | Yes | Yes | Yes | N/C | N/A | Yes |
| Kojić-Damjanov S | N/C | Yes | Yes | Yes | Yes | No | No | N/C | N/A | Yes |
| Li L (2018) | Yes | Yes | Yes | Yes | Yes | No | No | N/C | N/A | Yes |
| Li ZM | Yes | Yes | Yes | Yes | Yes | No | No | N/C | N/A | Yes |
| Li L (2022) | Yes | Yes | Yes | Yes | Yes | Yes | Yes | Yes | N/A | Yes |
| Li NN | Yes | Yes | Yes | Yes | Yes | Yes | Yes | Yes | N/A | Yes |
| Liu SQ | Yes | Yes | Yes | Yes | Yes | No | No | N/C | N/A | Yes |
| Liu ZJ | Yes | Yes | Yes | Yes | Yes | Yes | No | Yes | N/A | Yes |
| Rahman MM | Yes | Yes | Yes | Yes | Yes | Yes | Yes | Yes | N/A | Yes |
| Ma HY | Yes | Yes | Yes | Yes | Yes | No | No | Yes | N/A | Yes |
| Kucukazman M | Yes | Yes | Yes | Yes | Yes | No | No | Yes | N/A | Yes |
| Zhao MM | Yes | Yes | Yes | Yes | Yes | Yes | Yes | N/C | N/A | Yes |
| Eslami O | Yes | Yes | Yes | Yes | Yes | Yes | Yes | N/C | N/A | Yes |
| Ren L | N/C | Yes | Yes | Yes | Yes | Yes | Yes | Yes | N/A | Yes |
| Seo KI | Yes | Yes | Yes | Yes | Yes | No | No | Yes | N/A | Yes |
| Lim SH | Yes | Yes | Yes | Yes | Yes | Yes | Yes | Yes | N/A | Yes |
| Song Y | N/C | Yes | Yes | Yes | Yes | Yes | Yes | Yes | N/A | Yes |
| Sun Y | N/C | Yes | Yes | N/C | Yes | Yes | No | N/C | N/A | Yes |
| Sung KC | Yes | Yes | Yes | Yes | Yes | Yes | Yes | N/C | N/A | Yes |
| Wang L | Yes | Yes | Yes | N/C | N/C | Yes | Yes | N/C | N/A | Yes |
| Wen LN | Yes | Yes | Yes | Yes | Yes | No | No | Yes | N/A | Yes |
| Xu YH | Yes | Yes | Yes | Yes | Yes | No | No | Yes | N/A | Yes |
| Xu Z | Yes | Yes | Yes | Yes | Yes | Yes | No | Yes | N/A | Yes |
| Yuan JJ | N/C | Yes | N/C | Yes | Yes | No | No | Yes | N/A | No |
| Feng Y | Yes | Yes | Yes | Yes | Yes | Yes | Yes | N/C | N/A | Yes |
| Feng Z | Yes | Yes | Yes | Yes | Yes | Yes | Yes | N/C | N/A | Yes |
| Zhang L | Yes | Yes | Yes | Yes | Yes | No | No | Yes | N/A | Yes |
| Zhang LY | N/C | Yes | Yes | Yes | Yes | Yes | No | Yes | N/A | Yes |

Note: N/C, Unclear; N/A, Not applicable

**Supplementary Table 5 Sub-group analysis of different countries**

| **Variate** | **Number of studies** | **MD, 95% CI** | **Heterogeneity Test** | |
| --- | --- | --- | --- | --- |
|  |  |  | ***I*^2^ (%)** | ***P*** |
| **TC** |  |  |  |  |
| Ethiopia | 1 | 0.71 (0.48, 0.94) |  | ＜0.05 |
| Japan | 1 | 0.04 (0.04, 0.05) |  | ＜0.05 |
| Korea | 6 | 0.07 (0.04, 0.10) | 70.6 | ＜0.05 |
| Serbia | 1 | 0.83 (0.45, 1.21) |  | ＜0.05 |
| China | 26 | 0.21 (0.16, 0.25) | 97.4 | ＜0.05 |
| Saudi Arabia | 1 | 0.27 (0.11, 0.43) |  | ＜0.05 |
| Bangladesh | 1 | -0.16 (-0.39, 0.07) |  | ＞0.05 |
| Turkey | 1 | 0.39 (0.11, 0.67) |  | ＜0.05 |
| Iran | 1 | -0.05 (-0.21, 0.11) |  | ＞0.05 |
| Total | 39 | 0.15 (0.13, 0.17) | 96.5 | ＜0.05 |
| **TG** |  |  |  |  |
| Ethiopia | 1 | 1.22 (0.86, 1.58) |  | ＜0.05 |
| Japan | 1 | 0.07 (0.05, 0.09) |  | ＜0.05 |
| Korea | 6 | 0.08 (0.01, 0.15) | 72.5 | ＜0.05 |
| Serbia | 1 | 0.44 (0.07, 0.81) |  | ＜0.05 |
| China | 25 | 0.13 (0.09, 0.17) | 91.8 | ＜0.05 |
| Saudi Arabia | 1 | 0.37 (0.25, 0.49) |  | ＜0.05 |
| Bangladesh | 1 | -0.16 (-0.56, 0.24) |  | ＞0.05 |
| Turkey | 1 | 0.10 (-0.36, 0.56) |  | ＞0.05 |
| Iran | 1 | -0.13 (-0.38, 0.12) |  | ＞0.05 |
| Total | 38 | 0.13 (0.10, 0.16) | 90.5 | ＜0.05 |
| **LDL-C** |  |  |  |  |
| Ethiopia | 1 | 0.46 (0.27, 0.65) |  | ＜0.05 |
| Korea | 6 | 0.10 (0.07, 0.14) | 84.3 | ＜0.05 |
| Serbia | 1 | 0.46 (0.10, 0.83) |  | ＜0.05 |
| China | 25 | 0.20 (0.15, 0.26) | 98.9 | ＜0.05 |
| Saudi Arabia | 1 | 0.17 (0.04, 0.31) |  | ＜0.05 |
| Bangladesh | 1 | -0.20 (-0.39, -0.01) |  | ＜0.05 |
| Turkey | 1 | 0.31 (0.09, 0.53) |  | ＜0.05 |
| Iran | 1 | -0.07 (-0.14, 0.00) |  | ＞0.05 |
| Total | 37 | 0.18 (0.14, 0.22) | 98.5 | ＜0.05 |
| **HDL-C** |  |  |  |  |
| Ethiopia | 1 | 0.00 (-0.05, 0.05) |  | ＞0.05 |
| Japan | 1 | -0.07 (-0.07, -0.06) |  | ＜0.05 |
| Korea | 6 | -0.04 (-0.05, -0.03) | 74.0 | ＜0.05 |
| Serbia | 1 | 0.08 (-0.02, 0.18) |  | ＞0.05 |
| China | 27 | -0.02 (-0.03, -0.01) | 93.3 | ＜0.05 |
| Saudi Arabia | 1 | -0.15 (-0.20, -0.10) |  | ＜0.05 |
| Bangladesh | 1 | -0.01 (-0.05, 0.03) |  | ＞0.05 |
| Turkey | 1 | 0.05 (-0.05, 0.15) |  | ＞0.05 |
| Iran | 1 | 0.06 (-0.01, 0.13) |  | ＞0.05 |
| Total | 40 | -0.02 (-0.03, -0.01) | 97.2 | ＜0.05 |

**Supplementary Table 6 Sub-group analysis of different research types**

| **Variate** | **Number of studies** | **MD, 95% CI** | **Heterogeneity Test** | |
| --- | --- | --- | --- | --- |
|  |  |  | ***I*^2^ (%)** | ***P*** |
| **TC** |  |  |  |  |
| Cross-sectional study | 27 | 0.15 (0.12, 0.18) | 92.5 | ＜0.05 |
| Case-control study | 12 | 0.21 (0.13, 0.29) | 98.5 | ＜0.05 |
| Total | 39 | 0.15 (0.13, 0.17) | 96.5 | ＜0.05 |
| **TG** |  |  |  |  |
| Cross-sectional study | 27 | 0.08 (0.05, 0.12) | 87.7 | ＜0.05 |
| Case-control study | 11 | 0.21 (0.13, 0.29) | 94.2 | ＜0.05 |
| Total | 38 | 0.13 (0.10, 0.16) | 90.5 | ＜0.05 |
| **LDL-C** |  |  |  |  |
| Cross-sectional study | 25 | 0.16 (0.12, 0.20) | 92.5 | ＜0.05 |
| Case-control study | 12 | 0.19 (0.10, 0.29) | 99.4 | ＜0.05 |
| Total | 37 | 0.18 (0.14, 0.22) | 98.5 | ＜0.05 |
| **HDL-C** |  |  |  |  |
| Cross-sectional study | 28 | -0.02 (-0.04, -0.01) | 96.2 | ＜0.05 |
| Case-control study | 12 | -0.02 (-0.04, -0.00) | 95.1 | ＜0.05 |
| Total | 40 | -0.02 (-0.03, -0.01) | 97.2 | ＜0.05 |
